# A Newly Isolated Strain of *Haematococcus pluvialis* GXU-A23 Improves the Growth Performance, Antioxidant and Anti-Inflammatory Status, Metabolic Capacity and Mid-intestine Morphology of Juvenile *Litopenaeus vannamei*

**DOI:** 10.3389/fphys.2022.882091

**Published:** 2022-04-25

**Authors:** HaoHang Fang, ZhenXiao Zhuang, LuoDong Huang, Jin Niu, Wei Zhao

**Affiliations:** ^1^ College of Life Sciences, Sun Yat-sen University, Guangzhou, China; ^2^ Institute of Marine Research, Bergen, Norway; ^3^ College of Life Science and Technology, Guangxi University, Nanning, China

**Keywords:** *Haematococcus pluvialis* GXU-A23, *Litopenaeus vannamei*, growth performance, hepatopancreas health, metabolism, intestine morphology

## Abstract

*Haematococcus pluvialis* can be used as a green additive in aquafeeds due to it contains rich astaxanthin and polyunsaturated fatty acid. In the present study, a newly strain of *H. pluvialis* GXU-A23 with high concentration of astaxanthin was firstly isolated by a newly culture strategy in our laboratory. In addition, *H. pluvialis* GXU-A23 was applied in the *Litopenaeus vannamei* feed for determining whether it has positive effects on the growth performance, antioxidant and anti-inflammatory status, metabolic capacity and mid-intestine morphology of juvenile *L. vannamei*. Shrimp with 0.63 g approximately initial body weight were fed diets supplemented with/without 50 g/kg *H. pluvialis* GXU-A23. After 8 weeks feeding intervention, significantly higher growth performance of *L. vannamei* was obtained in the *H. pluvialis* GXU-A23 treatment group compared to the control group (*p* < 0.05). At the same time, *L. vannamei* fed with *H. pluvialis* GXU-A23 acquired significantly better antioxidant and anti-inflammatory status than the control group (*p* < 0.05). In addition, higher RNA expression level of hepatopancreas digestive enzyme, hepatopancreas lipid and glucose metabolic enzymes as well as better mid-intestine morphology were found in the *H. pluvialis* GXU-A23 treatment group than the control group (*p* < 0.05). These results indicated that 50 g/kg *H. pluvialis* GXU-A23 was suitable for the *L. vannamei* feed, which could improve the growth performance, antioxidant and anti-inflammatory status, metabolic capacity and mid-intestine morphology of juvenile *L. vannamei.*

## Introduction

The speedy development of aquaculture provided considerable high-quality protein for human (Costello et al., 2020; Cottrell et al., 2021). In fact, high production is attributed to high density farming (Bostock et al., 2010). On the other hand, many aquaculture environments were polluted due to the improvement of human activities (Zhang et al., 2019). However, these two factors might cause the growth of pathogen microorganisms, such as white spot syndrome virus (WSSV) (Verbruggen et al., 2016) and *Vibrio* parahaemolyticus (Soto-Rodriguez et al., 2015), in water and thus inducing the low survival rate of *Litopenaeus vannamei*, which severely limited the development of the shrimp industry. In order to reduce adverse effects of shrimp as caused by pathogen microorganisms, antibiotics were widely used in recent 20 years (Romero et al., 2012). However, limitations of antibiotic used in aquaculture are antibiotic resistance and drug residues (Sørum, 2005; Santos and Ramos, 2016). Therefore, to promote the development of aquaculture industry, proper green additives must be exploited for substituting the antibiotic used during the farming.

The flesh pigment is one of the essential factors which might influence the shrimp price since customers generally regard the optimal pigment as high quality (Diler and Gokoglu, 2004). However, crustaceans are unable to biosynthesize carotenoids *de novo*, while they can obtain and convert pigment from the feed into carotenoids and then deposit in the flesh (Niu et al., 2009). Therefore, optimization of the flesh pigment could be taken into consideration when it comes to exploiting a shrimp additive.

Astaxanthin, one of the keto carotenoids, is mainly existing in algae (like *Haematococcus pluvialis*, *Chlorella zofingiensis*), bacteria (like *Phaffia rhodozyma*) and crustaceans (Johnson and Lewis, 1979; Ip and Chen, 2005). The antioxidant property of astaxanthin was demonstrated more 100–500 folds than vitamin E to inhibit the lipid peroxidation *in vitro* (Ni et al., 2015). Dietary supplementation of astaxanthin bring many benefits to aquatic animals. For example, improving the growth performance (Wang et al., 2018), reducing the interval of molt cycle (Petit et al., 1997), enhancing the antioxidant and anti-inflammatory capacity (Xie J. et al., 2020), optimization of shrimp pigment (Ju et al., 2011).

Newly strain of *H. pluvialis* GXU-A23 with high concentration astaxanthin (33 g/kg) was isolated and cultured by a newly two-step batch culture strategy in our laboratory (Wang et al., 2019). In this method, modified Bold’s Basal medium (mBBM) (Wang et al., 2019) with 9.0 mM urea was provided to culture the *H. pluvialis* GXU-A23. Compared to the modified BG-11 medium (mBG-11) (Gao et al., 2016), the *H. pluvialis* could obtain remarkably higher astaxanthin content in the mBBM (Domınguez-Bocanegra et al., 2004; Nahidian et al., 2018). Apart from that, the *H. pluvialis* showed the better astaxanthin accumulation property in the urea as nitrogen source than NaNO_3_ and NH_4_HCO_3_ (Wang et al., 2019). In our previous study, the two-step batch culture strategy was used to successfully culture *H. pluvialis* JNU35, which contained 31.70 g/kg astaxanthin (Zhao et al., 2021). Since astaxanthin was mostly produced by nature *H. pluvialis*, the newly strain of *H. pluvialis* GXU-A23 could remarkably improve the production industry of astaxanthin as well as reduce the product budget. In addition, this microalga also could be used as a green additive in aquafeeds and beneficial for sustainable development of aquaculture.

The present study aims to evaluate whether there are beneficial effects of the newly isolated strain of *H. pluvialis* GXU-A23 on *L. vannamei.* Therefore, an 8 weeks feeding experiment was conducted to investigate effects of *H. pluvialis* GXU-A23 on the growth performance, antioxidant and anti-inflammatory status, metabolic capacity and mid-intestine morphology of juvenile *L. vannamei.* These results might provide a reference for feed formulation of *L. vannamei.*

## Materials and Methods

### *Haematococcus pluvialis* GXU-A23 Culture

*H. pluvialis* GXU-A23 was obtained from Zixi Mountain of Chuxiong (YunNan, China), and these microalgae were bacteria-free cultured in our laboratory. The culture method of *H. pluvialis* GXU-A23 was following the manuscript by Wang et al. (2019). Briefly, *H. pluvialis* GXU-A23 was scale-up cultured in a flat glass photobioreactor (length: 240 cm; height: 120 cm) with 6 cm light paths. mBBM (9.0 mM urea), bubbled gas of 1% CO2 (v/v) as well as 100 μmol/m^2^ s^−1^ continuous unilateral lighting were provided to culture the *H. pluvialis* GXU-A23 for 15 days. Afterward, *H. pluvialis* GXU-A23 was transferred into the same sized photobioreactor with nitrogen-free medium, 3 cm light paths and continuous bilateral illumination of 400 μmol m^−2^ s^−1^ for 15 days to accumulate astaxanthin. Afterward, red cells of *H. pluvialis* GXU-A23 were harvested by auto-precipitation and freeze-dried using freezing dryer. The freeze-drying biomass of *H. pluvialis* GXU-A23 contained 33 g/kg astaxanthin.

### Diet Preparation

As shown in Table 1, two isonitrogen and isolipids experimental diets were formulated with/without *H. pluvialis* GXU-A23 (D1: 0; D2: 50 g/kg) respectively. Dietary ingredients were purchased from Guangzhou Chengyi Company Ltd. (Guangzhou, China). The level of *H. pluvialis* GXU-A23 used in the present study was referred to our previous study (Zhao, et al., 2020), which was normally the highest concentration of additive used in the aquafeed.

**TABLE 1:** Ingredients and proximate compositions of two experimental diets (g/kg).

Ingredients	D1	D2
Fish meal	250	250
Soybean meal	270	270
Peanut meal	120	120
Wheat flour	234	184
Beer yeast	30	30
Shrimp bran powder	30	30
Fish oil	10	10
Soybean lecithin	10	10
Soybean oil	10	10
Choline chloride (50%)	5	5
Vitamin C phosphate	1	1
Vitamin and mineral premix^a^	20	20
Monocalcium phosphate	10	10
*Haematococcus pluvialis* GXU-A23 ^b^	0	50
Sum	1000	1000
Nutrient levels^c^(%)		
Moisture	7.45	7.63
Crude lipid	7.12	7.01
Crude protein	40.52	40.74
Ash	9.46	9.59
Astaxanthin	–	0.16

a Composition of vitamin and mineral mixture (kg^−1^ of mixture): vitamin A, 250,000 IU; riboflavin, 750 mg; pyridoxine HCL, 500 mg; cyanocobalamin, 1 mg; thiamin, 500 mg; menadione, 250 mg; folic acid, 125 mg; biotin, 10 mg; a-tocopherol, 3750 mg; myo-inositol, 2500 mg; calcium pantothenate, 1250 mg; nicotinic acid, 2000 mg; vitamin D_3_, 45,000 IU; vitamin C, 7000 mg, Zn, 4000 mg; K, 22.500 mg; I, 200 mg; NaCl, 2.6 g; Cu, 500 mg; Co., 50 mg; FeSO_4_, 200 mg; Mg, 3000 mg; Se, 10 mg.

b *Haematococcus pluvialis* GXU-A23: 33 g/kg astaxanthin (Dry matter).

c Measured values (Dry matter).

Measured nutrition values of diets were 7% crude lipid and 40% protein approximately (Table 1). The method of diet preparation was following the reported by Yu et al. (2016). Briefly, all dried ingredients and oils were weighted following table 1 and then completely homogeneous in the Hobart-type mixer (A-200T Mixer, Canada). Then, deionized water (250 ml/kg dried ingredients mixture) was added into the above ingredient to thoroughly mix for 15 min. Then, diets (1.2 mm diameters) were extruded using the pelletizer (South China University of Technology, China). Then, diets were heated in the 50°C ventilated oven for 120 min. Then, diets were stored at −20°C and kept away from the light until the feeding trial.

### Feeding Experiment

Juvenile *L. vannamei* were obtained and cultured at the experimental station of the Chinese Academy of Fishery Science (Lingshui, China). Before the trial, shrimp were acclimated to the experimental environment by feeding with D1 diet for 30 days 320 lively shrimp with an initial body weight of 0.63 g approximately were distributed randomly into the recirculating water system with eight cylindrical fiber tanks (300 L). Each of diets was randomly allocated to quadruplicate tanks. The feeding frequency was three times daily at 06:00, 12:00, and 18:00 with 8% of total shrimp weight and lasted for 8 weeks. During the period of feeding, environmental conditions were maintained as follows: water temperature: 26.8–28.1°C; pH: 7.5–7.7; salinity: 29—32‰; dissolved oxygen: > 7.0 mg/L; total ammonia nitrogen: < 0.1 mg/L; sulfide: < 0.05 mg/L. Natural light-dark (12–12 h) cycle was used during the feeding trial.

### Sample Collection

After 8 weeks feeding, *L. vannamei* were starved for 24 h. Then, all shrimp from each tank were weighed, counted and then recorded. Then, eight individuals from each tank were randomly collected and anesthetized (MS-222, 98%, Sigma, United States) for obtaining the blood sample. Then, hepatopancreas samples were removed for analysis of antioxidant parameters and mRNA expression; same sections of mid-intestine were removed and fixed in 4% paraformaldehyde (Beyotime, China) for intestinal histological examination. Blood samples were stored at the fridge (4°C, 12 h) and then centrifuged (7,100 g, 10 min, at 4°C) to obtain hemolymph for antioxidant parameters analysis. All hepatopancreas and hemolymph samples were separated rapidly and then maintained at −80°C until examination.

### Astaxanthin Analysis of *Haematococcus pluvialis* GXU-A23 and Feeds

Astaxanthin contents of *H. pluvialis* GXU-A23 and feeds were determined by spectrophotometrically as the description by Li, et al. (2012).

### Chemical Analysis of Feeds

Chemical compositions (moisture, crude lipid, crude protein and ash) of feeds were determined according to standard methods of AOAC (Horwitz, 2010). Briefly, moisture was analyzed by drying in the ventilated oven at 105°C until constant weight; crude lipid examination was performed following the Soxhlet extractor method (Soxtec System HT6, Tecator, Sweden); crude protein (N × 6.25) was measured following the Kjeldahl method (1030—Autoanalyzer; Tecator, Höganäs, Sweden); ash was analyzed using muffle furnace at 550°C until constant weight.

### Quantification of Hepatopancreas and Hemolymph Parameters Related to Antioxidant Status

Hepatopancreas were homogenized and centrifuged according to the description of Fang et al. (2021). Briefly, hepatopancreas were homogenized (1:9) in phosphate buffer. Afterward, above homogenates were centrifuged (10 min, 4°C, 1200 g) and then supernatants were collected.

Enzyme activities of total superoxide dismutase (T-SOD) (A001-1), total antioxidant capacity (T-AOC) (A015–2), glutathione peroxidase (GSH-PX) (A005-1) as well as the content of malondialdehyde (MDA) (A003-1) were measured according to instructions of reagent (Nanjing Jiancheng Bioengineering Institute, Nanjing, China) (Instructions of reagent were shown in additional files).

### Examination of Mid-intestine Histology

Mid-intestine sections were obtained and stained following the manuscript of Zhao et al. (2020). Briefly, tissue sections were stained using the hematoxylin and eosin (Beyotime, China), and mid-intestine histology were observed using the microscope (Olympus CKX41 microscope, Tokyo, Japan). The villus height and the mucosal layer thickness are equating to the average value of randomly selected eight villi and eight mucosal per slide respectively (Chen et al., 2020).

### mRNA Isolation and Expression Quantification

Hepatopancreas total RNA isolation and mRNA expression examination were performed following our previous manuscript (Fang et al., 2019). Briefly, the total RNA was isolated using Trizol^®^ reagent (Invitrogen, United States) following the manufacturer’s instruction. 1% agarose gel electrophoresis and spectrophotometer (NanoDrop 2000; Thermo Fisher, United States) were used to ascertain RNA quality and quantity, respectively. Afterward, cDNA was synthesized using the PrimeScript TM RT Reagent kit (Takara, Japan), following the manufacturer’s instruction. Real-time PCR for the target genes were performed using SYBR^®^ Premix Ex TaqTM II (Takara, Japan) and quantified on the LightCycler 480 (Roche Applied Science, Basel, Switzerland).

Primers related to the present study were listed in table 2. The elongation factor a (*ef1a*) was used as a housekeeping gene for RNA expression analysis (Guzmán-Villanueva et al., 2020). The relative mRNA expression of target genes was determined using the 2^−ΔΔCT^ method (Livak and Schmittgen, 2001).

**TABLE 2:** Sequences of primers used for real-time quantitative PCR.

Gene	Primer Sequence (5′-3′)	References
*ef1a*-F	TGG​CTG​TGA​ACA​AGA​TGG​AC	Xie et al. (2018)
*ef1a*-R	AGA​TGG​GGA​TGA​TTG​GGA​CC	
*sod*-F	CCG​TGC​AGA​TTA​CGT​GAA​GG	Duan et al. (2018)
*sod*-R	GTC​GCC​ACG​AGA​AGT​CAA​TG	
*gsh-px* F	GGC​ACC​AGG​AGA​ACA​CTA​C	Xie et al. (2018)
*gsh-px* R	CGA​CTT​TGC​CGA​ACA​TAA​C	
*cat*-F	TAC​TGC​AAG​TTC​CAT​TAC​AAG​ACG	Xie et al. (2019)
*cat*-R	GTA​ATT​CTT​TGG​ATT​GCG​GTC​A	
*relish*-F	CTA​CAT​TCT​GCC​CTT​GAC​TCT​GG	Xie et al. (2018)
*relish*-R	GGC​TGG​CAA​GTC​GTT​CTC​G	
*rho*-F	GTG​ATG​GTG​CCT​GTG​GTA​AA	Xie et al. (2018)
*rho*-R	GCC​TCA​ATC​TGT​CAT​AGT​CCT​C	
*chymotrypsin*-F	GGCTCTCTTCATCGACG	Xie J. et al. (2020)
*chymotrypsin*-R	CGTGAGTGAAGAAGTCGG	
*trypsin*-F	TCC​AAG​ATC​ATC​CAA​CAC​GA	Xie S. et al. (2020)
*trypsin*-R	GAC​CCT​GAG​CGG​GAA​TAT​C	
*hk*-F	AGT​CGC​AGC​AAC​AGG​AAG​TT	Yang et al. (2021)
*hk*-R	CGC​TCT​TCT​GGC​ACA​TGA​TA	
*fas*-F	GCG​TGA​TAA​CTG​GGT​GTC​CT	Yang et al. (2021)
*fas*-R	ACG​TGT​GGG​TTA​TGG​TGG​AT	

### Statistical Analysis

Experimental data in the present study are shown as means ± standard error (SE). Data were checked for normality and homogeneity of variance in the software of SPSS 22.0 (Chicago, United States) and then analyzed by independent-sample t-test. *p* < 0.05 was regarded as the significant difference between groups.

## Result

### Growth Performance and Feed Utilization

As shown in Table 3, dietary *H. pluvialis* GXU-A23 supplementation significantly altered the growth performance of *L. vannamei.* Significantly higher final body weight (FBW), weight gain rate (WGR) and specific growth rate (SGR) of *L. vannamei* were found in the D2 group than that of the D1 group (*p* < 0.05). However, dietary *H. pluvialis* GXU-A23 supplementation was unable to change the feed conversion ratio (FCR) of *L. vannamei* (*p >* 0.05). After 8 weeks feeding, survival rate (SR) of *L. vannamei* fed with/without *H. pluvialis* GXU-A23 were 96% approximately (*p >* 0.05).

**TABLE 3:** Growth performance and feed utilization of *L. vannamei* fed diet supplemented with/without *H. pluvialis* GXU-A23 for 56 days.

	D1	D2
IBW	0.64 ± 0.01	0.63 ± 0.01
FBW	5.98 ± 0.03[Table-fn Tfn4]	6.25 ± 0.01[Table-fn Tfn5]
WGR	828.31 ± 15.07[Table-fn Tfn4]	925.12 ± 14.84[Table-fn Tfn5]
SGR	3.98 ± 0.03[Table-fn Tfn4]	4.16 ± 0.03[Table-fn Tfn5]
FCR	1.24 ± 0.03	1.17 ± 0.01
SR	96.25 ± 1.25	96.88 ± 0.63

IBW (g per shrimp): initial body weight.

FBW (g per shrimp): final body weight.

Weight gain rate (WGR, %) = 100 * (final body weight—initial body weight)/initial body weight.

Specific growth rate (SGR, % day^−1^): 100× (Ln _final shrimp weight_ - Ln _initial shrimp weight_)/the experimental duration in days.

Feed conversion ratio (FCR) = dry diet fed/wet weight gain.

Survival rate (SR) (%) = 100 * (final number of shrimp)/(initial number of shrimp).

Values are mean ± SE (n = 4). Means in the same row with different superscripts are significantly different (*p* < 0.05).

### Oxidative Status Parameters

Antioxidant parameters of *L. vannamei* under different dietary intervention were shown in Table 4. Results showed that enzyme activities of hepatopancreas T-SOD, hepatopancreas GSH-PX as well as hemolymph T-SOD were significantly decreased in the D2 group than that in the D1 group (*p* < 0.05). Meanwhile, relatively lower hepatopancreas MDA content (*p > 0.05*) and remarkably lower hemolymph MDA content (*p* < 0.05) were found in the dietary *H. pluvialis* GXU-A23 supplementation group than the control group. No statistical differences of hepatopancreas T-AOC, hemolymph T-AOC and hemolymph GSH-PX were obtained between two experimental groups (*p > 0.05*).

**TABLE 4:** Hepatopancreas and hemolymph antioxidant status parameters of *L. vannamei* fed diet supplemented with/without *H. pluvialis* GXU-A23 for 56 days.

	D1	D2
Hepatopancreas		
T-SOD (U/mgprot)	10.4 ± 0.88^a^	5.61 ± 1.00^b^
T-AOC (U/mgprot)	0.27 ± 0.01	0.18 ± 0.03
GSH-PX (U/mg prot)	624.12 ± 49.36^a^	233.92 ± 56.78^b^
MDA (nmol/mgprot)	1.26 ± 0.03	1.1 ± 0.08
Hemolymph		
T-SOD (U/mL)	273.75 ± 6.08^a^	239.53 ± 9.52^b^
T-AOC (U/mL)	3.7 ± 0.12	3.66 ± 0.23
GSH-PX (U/mL)	419.35 ± 54.11	380.65 ± 19.36
MDA (mmol/ml)	8.27 ± 1.04^a^	3.84 ± 0.21^b^

Values are mean ± SE (*n* = 4).

Means in the same row with different superscripts are significantly different (*p* < 0.05).

### Hepatopancreas mRNA Expression Related to Immunity

**FIGURE 1:**
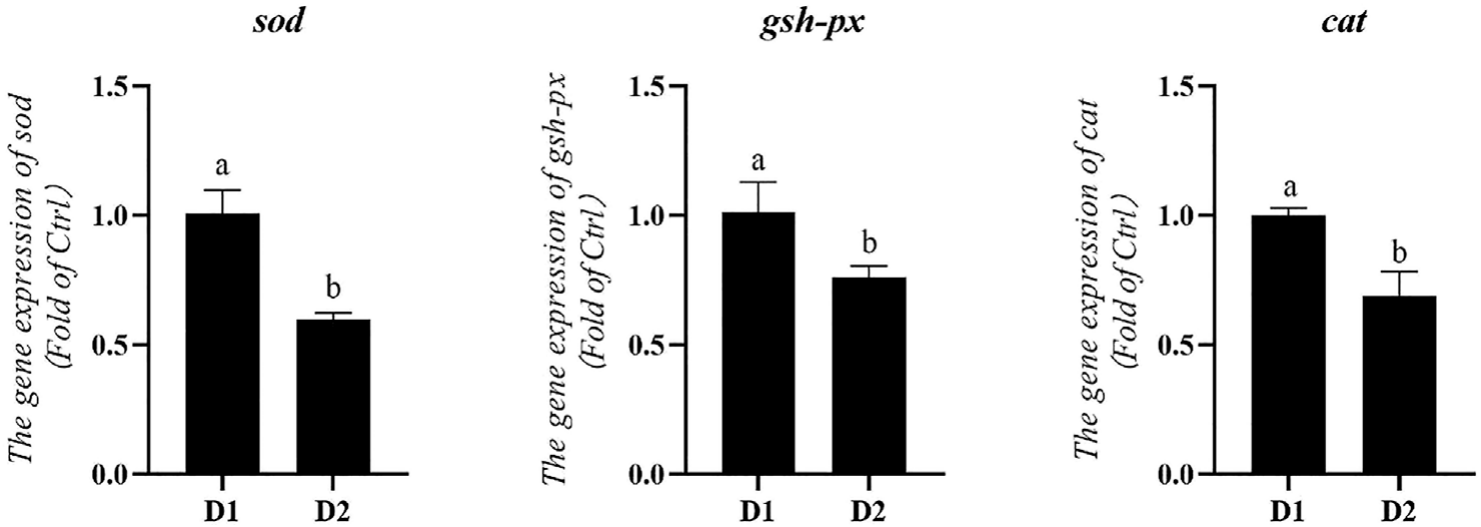
Hepatopancreas mRNA levels of antioxidative genes of *L. vannamei* fed diet supplemented with/without *H. pluvialis* GXU-A23 for 56 days. Values are mean ± SE (*n* = 4). The small letters indicated significant differences at *p* < 0.05.

mRNA expression levels of genes related to antioxidation of *L. vannamei* fed diet supplemented with/without *H. pluvialis* GXU-A23 were shown in Figure 1. Compared to the control group, the dietary *H. pluvialis* GXU-A23 supplementation group obtained significantly lower mRNA expression levels of *sod*, *gsh-px* and *cat* (*p* < 0.05).

**FIGURE 2:**
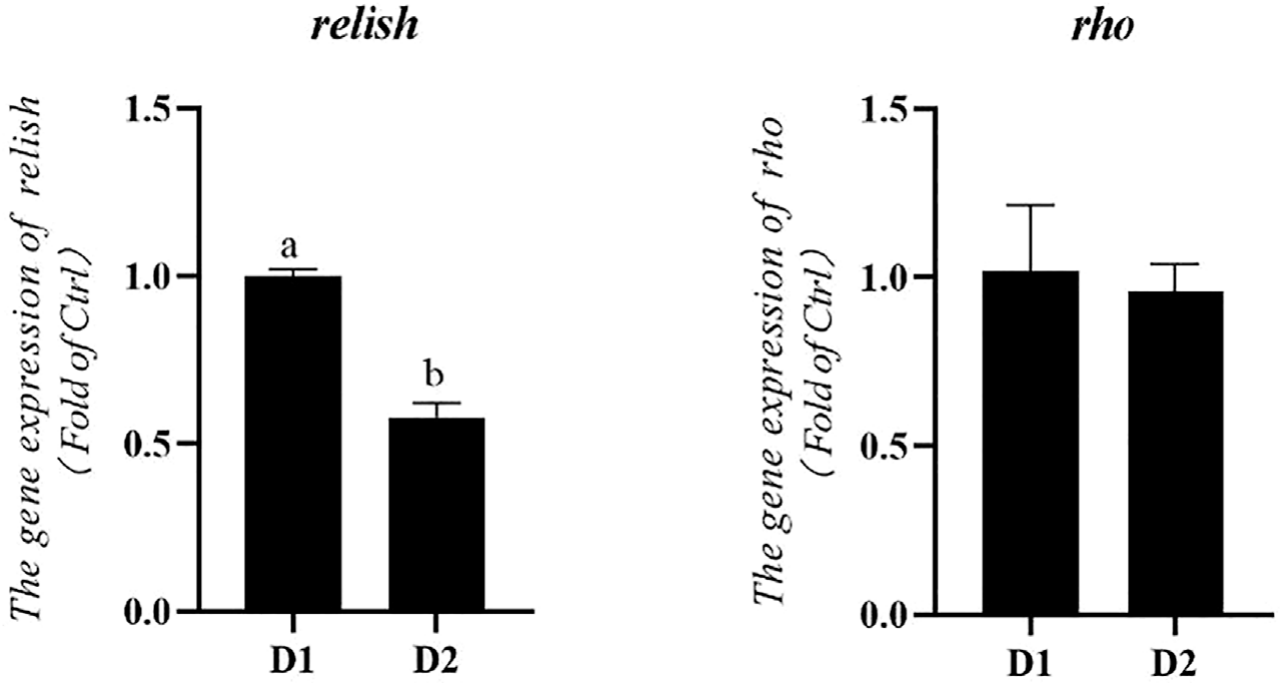
Hepatopancreas mRNA levels of anti-inflammatory genes of *L. vannamei* fed diet supplemented with/without *H. pluvialis* GXU-A23 for 56 days. Values are mean ± SE (*n* = 4). The small letters indicated significant differences at *p* < 0.05.

mRNA expression levels of anti-inflammatory genes of *L. vannamei* fed diet supplemented with/without *H. pluvialis* GXU-A23 were shown in Figure 2. Remarkably lower mRNA expression level of relish was obtained in the D2 group compared to the control group (*p* < 0.05). No statistical difference of the *rho* mRNA expression level was observed between two groups (*p* > 0.05).

### Hepatopancreas mRNA Expression Related to Digestive and Metabolic Enzymes

**FIGURE 3:**
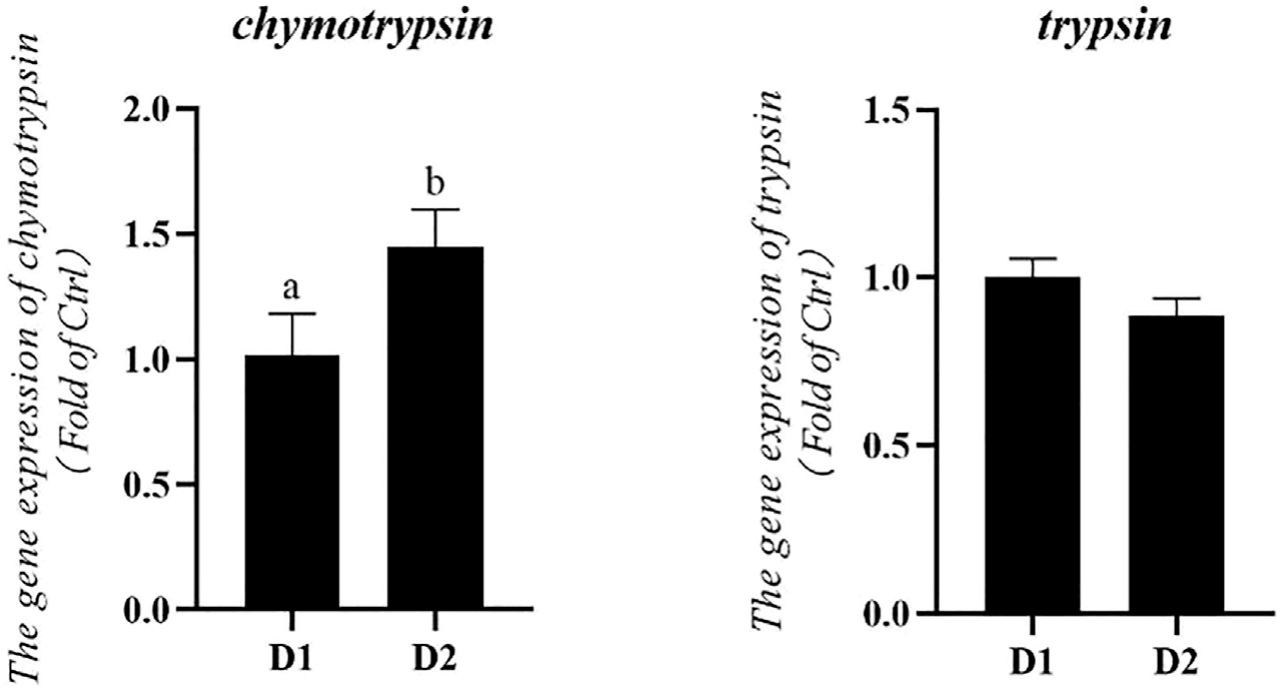
Hepatopancreas mRNA levels of digestive enzyme genes of *L. vannamei* fed diet supplemented with/without *H. pluvialis* GXU-A23 for 56 days. Values are mean ± SE (*n* = 4). The small letters indicated significant differences at *p* < 0.05.

mRNA expression levels of digestive enzymes of *L. vannamei* fed diet supplemented with/without *H. pluvialis* GXU-A23 were shown in Figure 3. The mRNA expression level of *chymotrypsin* of *L. vannamei* was significantly increased after dietary *H. pluvialis* GXU-A23 intervention (*p* < 0.05). However, no statistical difference of *trypsin* mRNA expression level was observed between two groups (*p* > 0.05).

**FIGURE 4:**
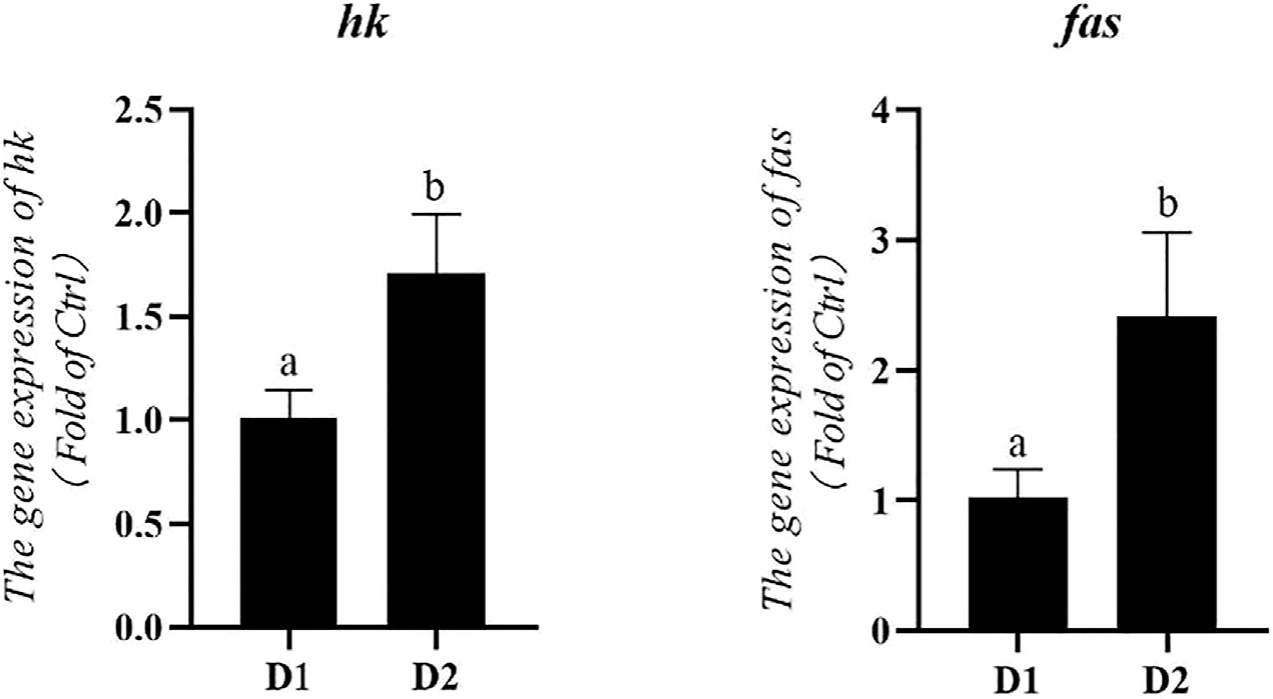
Hepatopancreas mRNA levels of metabolic enzymes genes of *L. vannamei* fed diet supplemented with/without *H. pluvialis* GXU-A23 for 56 days. Values are mean ± SE (*n* = 4). The small letters indicated significant differences at *p* < 0.05.

Dietary *H. pluvialis* GXU-A23 supplementation significantly altered the mRNA expression level of metabolic enzymes of *L. vannamei* (Figure 4). mRNA expression levels of *hexokinase* (*hk*) and *fatty acid synthase* (*fas*) were significantly higher in the *H. pluvialis* GXU-A23 treatment group compared to the control group (*p* < 0.05).

### Light Microscopy Observation of Mid-intestine Morphology

**FIGURE 5:**
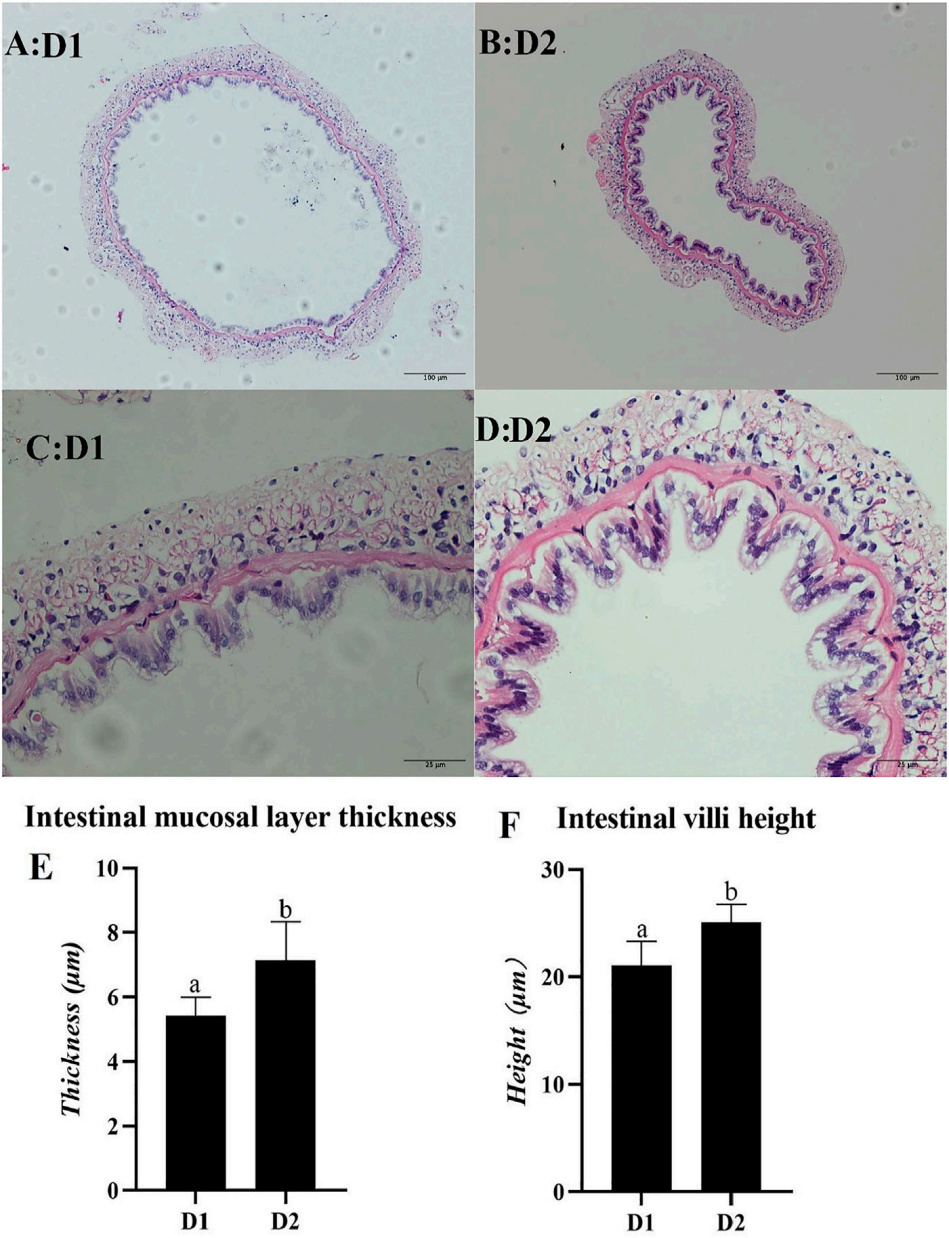
Light microscopy of mid-intestine morphology of *L. vannamei* fed diet supplemented with/without *H. pluvialis* GXU-A23 for 56 days. The scale bars of picture **(A,B)** were 100 μm, while the scale bars of picture **(C,D)** were 25 μm respectively. Picture **(E,F)** represents the intestinal mucosal layer thickness and intestinal villi height of *L. vannamei* respectively. Values are mean ± SE (*n* = 4). The small letters indicated significant differences at *p* < 0.05.

Light microscopy of mid-intestine morphology of *L. vannamei* exposed to different dietary treatment for 56 days was shown in Figure 5. Results showed that the intestinal mucosal layer thickness and villa height of *L. vannamei* fed with *H. pluvialis* GXU-A23 was significantly higher than that of the control group (*p* < 0.05).

## Discussion

In recent years, microalgae had been gained widely attention in aquafeeds due to it is the green additive with high nutrition (Roy and Pal, 2015). Different microalgae might contain different nutrients, such as high lipid and protein (Aaronson et al., 1980; Webb, 1983), proper amino acid pattern (Becker, 2004), polysaccharide (Chu et al., 1982; Lama et al., 1996), pigments (Metting, 1996) and vitamins (Brown and Farmer, 1994). Supplementation of microalgae in aquafeed can partly substitute for minerals (Fabregas and Herrero, 1986), fishmeal and fish oil (Shah et al., 2018). Microalgae as an aquafeed additive for improving the growth performance and immunity of animals was also widely reported (Cerezuela et al., 2012; Reyes-Becerril et al., 2013, 2014).

In the present study, *L. vannamei* fed with the *H. pluvialis* GXU-A23 diet obtained the better growth performance (WG and SGR) compared to that of the control group. Similar results also reported in *Pseudosciaena crocea* (Li et al., 2014)*, Trachinotus ovatus* (Zhao et al., 2021)*, L. vannamei* (initial weight: ∼ 1.0 g) (Ju et al., 2012). *H. pluvialis* GXU-A23 contains astaxanthin with 3S-3′S type which is the same structure in *Salmo salar* and other aquatic animals (Higuera-Ciapara et al., 2006). The main reason for astaxanthin could improve the growth performance of aquatic animals is that this pigment could mediate intermediate metabolism, resulting in enhancing nutrients utilization and thus optimization of the growth performance of *L. vannamei* (Han et al., 2018). However, *H. pluvialis* was unable to alter the growth performance in post-larval *L. vannamei* (5 days after metamorphosing of mysis stage) (Xie et al., 2018)*, L. vannamei* (initial weight: 0.94–0.99 g) (Ju et al., 2011) and *Cichlasoma citrinellum* (Pan and Chien, 2009). These different results might be attributed to the source and dose used of dietary *H. pluvialis,* the growth stage of animals as well as the experimental environment. Besides, the hepatopancreas mRNA expression level of *chymotrypsin* was upregulated in the *H. pluvialis* GXU-A23 feeding group than that of the control group. High expression of protease could improve the digestion and absorption of protein, and thus enhancing growth (Zokaeifar et al., 2012). Apart from digestive enzyme, the intestine morphology was strongly contributed to the growth of shrimp. Higher intestinal villi height represented the larger contact surface area between the intestine and nutrients (Emami et al., 2012), and the increasing of intestinal mucosal layer thickness meaning the improvement digestion and absorption ability of shrimp (Chen et al., 2020). In the present study, remarkably higher intestinal villi height and intestinal mucosal layer thickness were found in the dietary *H. pluvialis* GXU-A23 treatment group compared to the control group, indicating that *H. pluvialis* GXU-A23 has protective effect on mid-intestine morphology of *L. vannamei* and thus improves the growth performance of shrimp, which is consistent with the present result.

Generally, aquatic animals have the poor glucose utilization capacity because of the low level of insulin released (Chen et al., 2020). However, glycolysis is the only pathway of glucose metabolism in animals (Li et al., 2018). Among them, hepatic HK was a fundamental limitation enzyme in the glycolysis process (Lu et al., 2018). In the present study, the *H. pluvialis* GXU-A23 feeding *L. vannamei* group obtained higher hepatopancreas mRNA expression level of *hk* than the control group, indicating that dietary *H. pluvialis* GXU-A23 supplementation could improve the utilization capacity of blood glucose for satisfying higher energy requirement. Apart from glucose metabolism, lipid metabolism also plays a major role in health of aquatic animals. In particular, FAS plays an essential role in lipogenesis by catalyzing the *de novo* biosynthesis of fatty acids (Lu et al., 2018). In the present study, higher mRNA expression level of *fas* in the *L. vannamei* fed with *H. pluvialis* GXU-A23 group than that in the control group, indicating dietary *H. pluvialis* GXU-A23 supplementation was beneficial for the synthesis of hepatopancreas fatty acids.

When shrimp was subjected to environmental pressures, the breathing burst would be occurred to produce reactive oxygen species (ROS) for attacking invading microorganisms (Zhao et al., 2020). However, overproduction ROS might attack normal cells and then cause oxidative damages to shrimp. To avoid the riskiness of ROS, cells have developed an antioxidant system which involve various antioxidant enzymes, like SOD, GSH-PX, CAT (Zhao et al., 2017). In the present study, significantly lower antioxidant enzyme activities (hepatopancreas T-SOD, hepatopancreas GSH-PX and hemolymph T-SOD) as well as hepatopancreas mRNA expression levels (*sod*, *gsh-px* and *cat*) were obtained in dietary *H. pluvialis* GXU-A23 treatment group compared to the control group. Lower antioxidant parameters in the D2 group was attributed to the astaxanthin in *H. pluvialis* GXU-A23, which contains the ionone ring with hydroxyl and keto and thus it could scavenge ROS in crustaceans (Ambati et al., 2014). As a result, *L. vannamei* was unnecessary to produce more antioxidant enzymes. MDA is a lipid peroxidation product which is generally regarded as an essential parameter to evaluate the oxidative damage of animals (Larbi Ayisi et al., 2018). In the present study, *L. vannamei* fed with *H. pluvialis* GXU-A23 diet obtained the remarkably lower hemolymph MDA compared to the control group, indicating *H. pluvialis* GXU-A23 could prohibit the lipid peroxidation of cells and enhance the antioxidant capacity of *L. vannamei*.

Except for the antioxidant system, aquatic animals also responses to environmental stresses by regulating inflammatory responses (Fazelan et al., 2020). If subjected to stress, inflammatory mediators (like cytokines or prostaglandins) would be produced in cells for mediating the inflammatory system to remove detrimental irritations (Boltana et al., 2018). However, excessive inflammation response might lead to various pathological diseases, such as fever (Evans et al., 2015), loss of tissue function (Takeuchi and Akira, 2010). NF-κB signal pathway is closely correlated with the pathogenesis of inflammatory diseases (Yu et al., 2020). Among them, relish was a key NF-κB family protein in *L. vannamei* (Qiu et al., 2014). In the present study, the mRNA expression level of relish in the dietary *H. pluvialis* GXU-A23 supplementation group was significantly higher than that of the control group, indicating *H. pluvialis* GXU-A23 have a positive effect on inhibiting the NF-κB singal pathway. The prohibition of NF-κB pathway might narrow the production of pro-inflammatory cytokines, resulting in mitigating inflammatory responses (Xie et al., 2011). Therefore, *H. pluvialis* GXU-A23 plays an important role in alleviating inflammatory responses of *L. vannamei.*

## Conclusion

Overall, our present study demonstrated that dietary *H. pluvialis* GXU-A23 supplementation enhanced the growth performance of *L. vannamei* by improving antioxidant and anti-inflammatory status, metabolic metabolism and mid-intestine morphology. Therefore, 50 g/kg *H. pluvialis* GXU-A23 was recommended for the *L. vannamei* feed.

## Data Availability

The original contributions presented in the study are included in the article/Supplementary Material, further inquiries can be directed to the corresponding authors.
